# Non-pharmacological Approaches for Headaches in Young Age: An Updated Review

**DOI:** 10.3389/fneur.2018.01009

**Published:** 2018-11-27

**Authors:** Frank Andrasik, Licia Grazzi, Emanuela Sansone, Domenico D'Amico, Alberto Raggi, Eleonora Grignani

**Affiliations:** ^1^Department of Psychology, University of Memphis, Memphis, TN, United States; ^2^Neuroalgology Division, Fondazione IRCCS Istituto Neurologico Carlo Besta, Milan, Italy; ^3^Neurology, Public Health and Disability Unit, Fondazione IRCCS Istituto Neurologico Carlo Besta, Neurology, Milan, Italy

**Keywords:** cognitive-behavioral therapy, biofeedback, mindfulness, transcranial magnetic stimulation, migraine, tension-type headache, disability, depression

## Abstract

Headache disorders are common in children and adolescents. Most of the studies on non-pharmacological treatments have however been carried out on adults. In this review we provide information on recent studies examining non-pharmacological approaches for managing headache in children and adolescents. Our search of SCOPUS for primary studies conducted between January 2010 and July 2018 uncovered 11 controlled studies, mostly addressing behavioral approaches, in which a total of 613 patients with a diagnosis of primary headache, and average age 10.2–15.7 years (30–89% females) were recruited. Non-pharmacological treatments were shown to produce sizeable effects on the classical primary endpoint, i.e., headache frequency, with reductions from baseline ranging between 34 and 78%. Among commonly reported secondary endpoints, particularly disability, quality of life, depression and anxiety, marked improvements were noted as well. Taken as a whole, our findings suggest that non-pharmacological treatments constitute a valid option for the prevention of primary headaches in young age. Future research with higher-quality studies is needed. Particular attention needs to be given to studies that randomize patients to condition, blind researchers in charge of evaluating treatment outcomes, routinely include headache frequency as the primary endpoint, include adequate-length follow-up, address changes in biomarkers of disease and other possible mediators of outcome, and that employ predictive models to enhance the level of evidence for these approaches.

## Introduction

Headache disorders are common in children and adolescents, affecting up to 88% of the pediatric and adolescent population, with chronic headache types impacting up to 6% (1, 2). Headache can result in significant disability, including missed school days and limitations in extracurricular activities, such as social events with peers, family gatherings, and sports. Pharmacological treatment for acute episodes typically include non-steroidal anti-inflammatory drugs (NSAIDs), analgesics, and triptans. As with adults, appropriate administration is needed in order to be effective, with specific attention being given to providing information about the risk for medication overuse headache (3). Among preventive drugs, antiepileptics such as topiramate, are considered as first-line treatment (4), and several drugs used in the prevention of migraine in adults are commonly prescribed for children (5, 6). Side effects of particular relevance for children and adolescents include weight loss or weight gain, paresthesias, cognitive slowing, and sleepiness. Caution is warranted in adolescent females in particular due to the elevated risk of developing polycystic ovarian syndrome as well as possible teratogenic effects of many of these compounds. Drug treatment, however, is not always needed, and prophylactic treatment is not considered the first line treatment in the vast majority of cases (6, 7). In recent years, attention has been increasingly paid to non-pharmacological treatments of headache disorders, chiefly those that are cognitive, behavioral, or psychophysiological in nature, but with some attention to non-invasive neurostimulation (8–11). Overall, significant benefits, typically ranging from 35 to 50%, have been reported for the above-mentioned treatments with respect to reductions in headache frequency. However, most of the published studies on non-pharmacological treatments have been carried out on adults, and more recent literature reviews have not focused extensively on young headache patients. The aim of the present review is to help fill this gap by providing updated information on more recent investigations of non-pharmacological approaches to the treatment of headache in children and adolescents.

## Methods

### Search strategy

We performed a comprehensive search on SCOPUS covering the period January 2010–July 2018 to identify primary research papers reporting either randomized clinical trials (RCTs) or observational studies that addressed non-pharmacological approaches for headaches disorders in children and adolescents. The following combinations of key-words were searched within the titles, abstracts, or key-words provided:

- headache OR “tension type headache” OR migraine OR “chronic tension type headache” OR “chronic migraine” OR “medication overuse headache.”
- young OR adolesc^*^ OR juvenile.
- “cognitive behavio^*^ therapy” OR “acceptance and commitment therapy” OR ACT OR mindfulness OR biofeedback OR “relaxation training” OR “lifestyle modification^*^” OR “complementary alternative medicine” OR neuromodulation OR neurostimulation OR “single pulse transcranial magnetic stimulation” OR “repetitive transcranial magnetic stimulation” OR “transcutaneous supraorbital nerve stimulation” OR “non-invasive vagal nerve stimulation” OR “caloric vestibular stimulation” OR “sphenopalatine ganglion stimulation” OR “occipital nerve stimulation.”
- Our search was limited to original studies, published in English language peer-reviewed journals, and filtered by the following subject areas: Medicine, Neurosciences, Health Profession, Pharmacology, Toxicology and Pharmaceutics, Biochemistry, Genetics and Molecular Biology and Psychology. Finally, we filtered for other key-words clearly not germane to our topic (the detailed search strategy is included in Supplementary Materials).

### Inclusion and exclusion criteria for articles selected

We specifically searched for clinical trials and observational studies, either cross-sectional or longitudinal, and excluded reviews, commentaries, letters to the editors, editorials, qualitative studies, case reports and small case series (< 10 subjects).

To be included papers needed to provide sufficient information to extract the following: impact of non-pharmacological treatment on headache frequency or other outcomes, such as disability or quality of life, as assessed by patient-reported outcomes measures (PROMs) and/or parent report. Studies further had to focus on the primary headache disorders of migraine or tension-type headache. Studies drawing from populations that included other types of headache disorders (mixed disorders), or wherein the presence of headache was addressed chiefly as a symptom in the context of other general medical conditions were excluded. Finally, studies that included both adolescents and young adults were excluded if the findings were reported in aggregate and it was not possible to disentangle the outcomes for the adolescents or if the average age of the sample suggested that the study was predominantly carried out in a population of adults.

### Paper selection and data extraction

Selected abstracts were screened by a single researcher (EG) and, in order to ensure quality and consistency of data extraction, 20% of the abstracts along with the full texts were randomly selected for a second evaluation conducted by another reviewer (AR or ES) who was blind to the initial decision. We determined at the outset if agreement rates were below 70%, each of the double-checked abstract or manuscript would be re-reviewed by the two researchers to arrive at a final decision by consensus (however, as will be seen below, this did not surface as a problem).

Extracted information included the kind of non-pharmacological approach employed, broadly defined in terms of Cognitive Behavioral Therapy (CBT), a mindfulness-based approache, Biofeedback (BFT) treatment, Transcranial Magnetic Stimulation (TMS) and Multimodal treatment. We also recorded the main characteristics of selected studies, which included sample size, percentage of females, mean age, headache frequency at baseline and at follow-up, percent reduction and, when available, clinical significance of outcomes. We converted reported values for headache frequency to conform to a standard, comparable monthly period when authors described it differently (e.g., on a 3-month basis).

## Results

**Figure 1:**
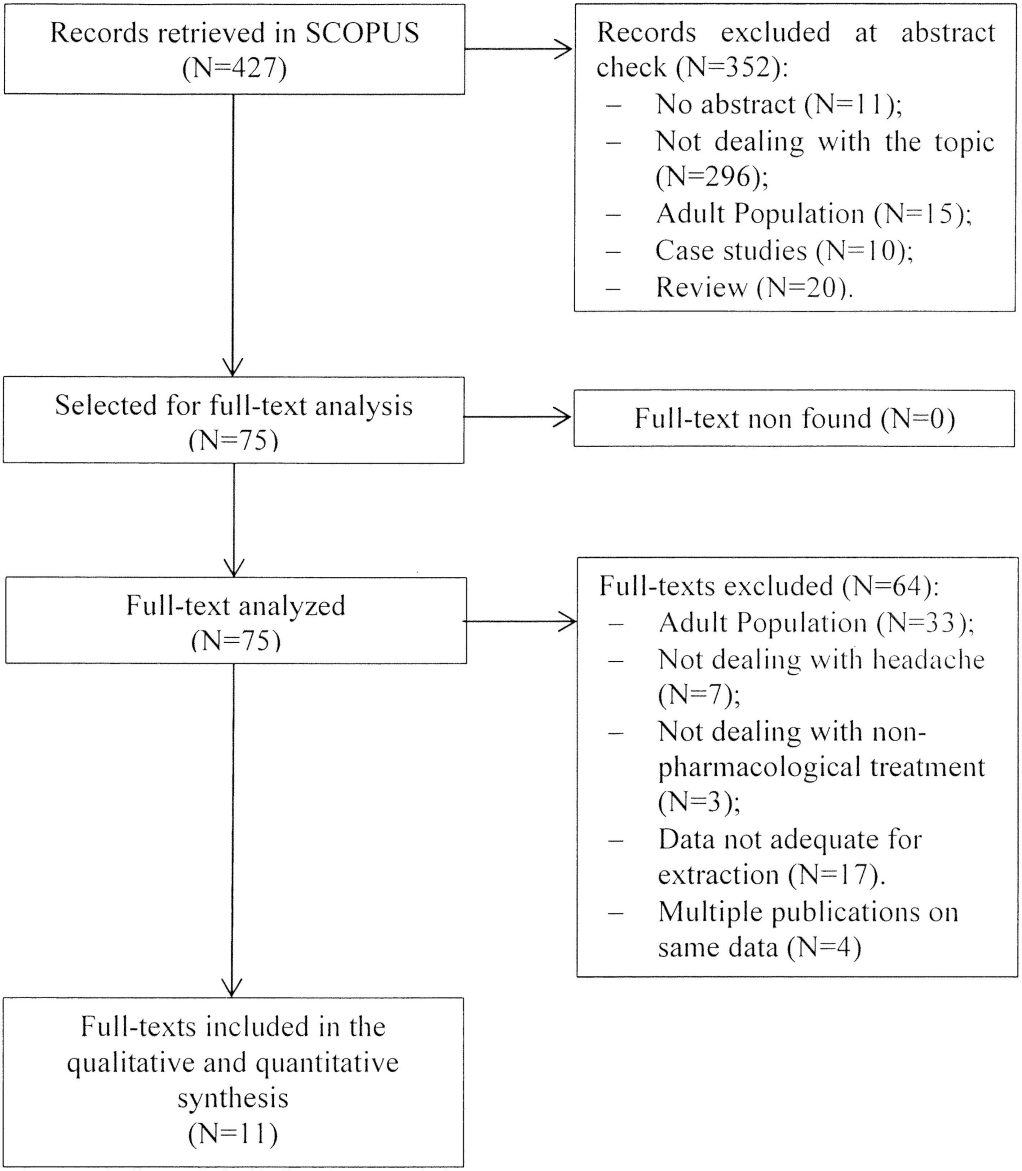
Flowchart of papers' selection.

The initial search returned 427 records. Following abstract screening and full text assessment, 11 publications were selected for inclusion in this review (12–22). The rate of agreement between reviewers was 99.5% at the abstract check, and 100% at full-text check. Figure 1 shows the PRISMA flow diagram of our search process. Table 1 presents a summary of the main outcome of the publications included in this review.

**Table 1:** Main characteristics and main outcomes of the included studies.

**ID**	**Treatment**	**Control**	**Headache type**	**Sample size (% W)**	**Age**	**Baseline HA freq—FU freq**	**% reduction (sig.)**	**Main results**
Powers et al. (12)	CBT + Amitriptyline	Headache education + Amitriptyline	CM	135 (80%)	14.4	21.4–6	−71.9%(^**^)	CBT determined significant reduction in headache frequency and improvements in migraine-related disability.
Rapoff et al. (13)	CBT	Medical therapy (as prescribed for usual clinical practice)	EM	35 (88%)	10.2	13.7–7.1	−48.2% (ns)	CBT determined improvements in post-intervention headache severity and in 3-months post-intervention QoL.
Hickman et al. (14)	CBT	Headache education	CH	36 (81.2%)	15.1	nr—nr		CBT determined significant improvements in headache disability, anxiety, depression and healthy lifestyle beliefs.
Law et al. (15)	Internet-delivered CBT	Specialized headache treatment (medical, physical, psychological)	Mixed (EM, TTH, both, other)	83 (81.9%)	14.5	24–15.4	−35.8% (^**^)	CBT determined significant headache reduction and improvements on headache pain intensity, activity limitations, depressive symptoms and parent protective behaviors.
Sharma et al. (16)	CBT	Medical therapy (as prescribed for usual clinical practice)	Mixed (Primary headache)	63 (47.6%)	13.91	nr—nr		CBT determined significant improvements on headache severity, clinician-rated overall anxiety and state and trait anxiety.
Tornoe and Skov (17)	BFB + RT	NA	Mixed (TTH/CTTH)	9 (88.9%)	10.9	19.1–11.1	−41.88% (^*^)	BFB determined significant headache frequency reduction.
Blume et al. (18)	BFB	NA	Mixed (Primary headache)	132 (69.7%)	13.4	14–8	−42.9% (^**^)	BFB determined improvements in headache frequency and severity.
Shiri et al. (19)	BFB	NA	Mixed (CTTH, CM)	10 (30%)	13.4	17—nr		BFB significantly improved ratings of pain, daily functioning and quality of life.
Hesse et al. (20)	Mind	NA	Recurrent head	15 (100%)	14.15	4.6—nr		Mindfulness determined improvements in depression symptoms, pain acceptance and in parents-rated physical health-related quality of life.
Irwin et al. (21)	TMS	NA	CM	12 (66.7%)	15	13.3–8.8	−33.8% (^*^)	TMS determined improvements in headache frequency, acute medication use and headache-related disability.
Prezekop et al. (22)	Multimodal treatment	Medical prophylaxis (Amitriptyline or Gabapentin)	CTTH	83 (80.7%)	15.7	22.3–4.9	−78% (^**^)	Multimodal treatment determined improvements in headache frequency, pain intensity, general health, pain restriction and number of bilateral tender points.

Significance was reported as

* p < 0.05;

** *p < 0.001; ns, not significant; nr, not reported*.

Across the studies, 613 participants with a diagnosis of primary headache and average age ranging between 10.2 and 15.7 years were recruited. Patients were mostly females, ranging on average between 30 and 88.9%, with one study including females only (20). Six RCTs involved samples of patients with different primary headaches; two studies involved patients with chronic migraine, one with episodic migraine, one with chronic tension type headache, one describing patients only as “chronic headache,” and one addressing “recurrent headache.”

Five papers consisted of single group studies, while the remaining six were RCTs in which a given non-pharmacologic treatment was compared with either treatment as usual (TAU) (13, 16), headache education (14, 15), education plus amitriptyline (12), amitriptyline or gabapentin (22). The majority of the studies evaluated the effects of CBT (12–16). Three studies included BFB treatment (17–19). The remaining three studies evaluated mindfulness (20), single-pulse TMS (21) and a multimodal treatment (22).

### Studies on CBT

Five RCTs assessed CBT, comparing it to education (13, 14), TAU (15, 16) or amitriptyline plus education (12). Two of these studies focused on adolescents experiencing different forms of primary headache (15, 16), whereas the other three trials evaluated CBT in patients with episodic migraine (13), chronic migraine (12) or “chronic headache” (14) alone. In all studies, CBT sessions were delivered weekly, for periods varying between 4 and 12 weeks.

CBT yielded significant reductions in headache frequency that ranged between 35.8 and 71.9% in two studies (12, 15). Although Rapoff and colleagues did not report a statistically significant reduction in headache frequency, the magnitude of improvement was sizeable 47.9%. Two trials did not report on change in headache frequency (14, 16).

Three of the above-mentioned studies (12–14) reported significant reductions in disability that ranged between 11.8 and 88.1%, as measured by the PedMIDAS. Other studies noted improvements in other secondary outcomes measured by PROMs investigating pain intensity (13, 15), quality of life (13), and parent protective behaviors, which include all parents' responses that, on one hand serve to reinforce pain complaints through increased parental attention and presence and, on the other hand, inappropriately lessen pain complaints by permitting children and adolescent to escape or avoid unwanted responsibilities or roles (15). Furthermore, the two studies that did not report on headache frequency focused their attention on other aspects that are often associated with headache, chiefly symptoms of anxiety and depression. The first of these two studies (14) evaluated the effects of a CBT intervention that focused on improving mental health overall (i.e., decrease perceived stress, anxiety and depression, while strengthening beliefs in ability to manage pain and to engage in a healthier lifestyle) and providing education about identifying and managing headache triggers. This 7-week treatment was compared to an education program of the same duration that focused on potential headache triggers (i.e., lifestyle, environmental, medication, hormonal, and dietary triggers) and headache hygiene measures (i.e., regular sleep and eating habits, moderate exercise, good hydration, and avoidance of caffeine, ethyl alcohol and other drugs). CBT produced significant reductions for symptoms of anxiety (11.3%) and depression (13.9%), as well as improvements with regards to headache disability and healthy lifestyle beliefs, when compared to headache education alone. In the second study, Sharma et al. (16) enrolled adolescents diagnosed as migraine or tension type headache, with comorbid anxiety disorders, who were randomized to either a transdiagnostic group CBT or a TAU control group. The intervention consisted of 12 weekly sessions that focused on identification of shared mechanisms across disorders, psychoeducation about headache and anxiety, cognitive restructuring, and stress management techniques. Adolescents within the CBT group showed significant improvements on headache severity and anxiety as assessed by clinical evaluations and PROMs.

### Studies on BFB treatment

Three single group outcome studies investigated various forms of BFB. The first study (17) included a sample of children with frequent or chronic tension-type headache who underwent 9 sessions of electromyographic biofeedback combined with computer animated relaxation therapy. Between baseline and 3-month follow-up, headache frequency decreased significantly, dropping from 19.1 to 11.1 headache days per month (49.1%). Furthermore, pericranial tenderness was significantly reduced among those who experienced frequent tension-type headache.

The second study, carried out by Blume et al. (18), involved children with different types of primary headaches, who underwent an average of 7 hand warming BFB sessions. Between baseline and the last training session, participants showed a significant reduction of 42.9% in headache frequency (decreasing from 14 to 8 headache/days per month). Median headache intensity also decreased significantly from a value of 6 at baseline to 5 at the final visit on a 10-point scale (16.7%).

Finally, Shiri et al. (19) evaluated the effects of a virtual reality system combined with BFB on a sample of children diagnosed with varied primary headaches. At the beginning of the treatment, participants had their picture taken in various emotional states to which they attached images representing their pain. During the 10 BFB sessions, children were instructed to watch their image and try to relax. Biofeedback yielded significantly improved ratings of pain by 51.9%, daily functioning by 67.4%, and quality of life by 20%. Moreover, the authors reported that most patients seemed to harness their new relaxation skills to relieve headache outside of the laboratory setting.

### Mindfulness-based intervention

Hesse et al. (20) evaluated a mindfulness-based intervention in a sample composed entirely of female adolescents experiencing “recurrent headaches.” All participants underwent eight 2 h weekly mindfulness sessions and were instructed to practice learned techniques at least once per day. The intervention was tailored to address headache and the resultant related distress by teaching the adolescents to become more mindful of breath and sounds, which was supplemented with didactic lessons and group discussions. Due to the small number of adolescents providing headache daily diaries, no formal analyses of improvements for headache frequency and severity were performed. However, improvements were noted with respect to depression symptoms (21.6% lower) and pain acceptance (22.2% lower). Further, while parent-rated questionnaires showed improved physical health-related quality of life (13.4%), reports by the adolescents did not reveal any meaningful decreases in disability over time. Although by no means definitive, this study suggests that mindfulness can be a feasible and acceptable intervention for adolescents with recurrent headaches.

### Transcranial magnetic stimulation (TMS)

One study investigated the efficacy of single-pulse TMS in adolescents diagnosed with chronic migraine (21). During the 12-week treatment period participants were instructed to apply the device twice daily, administering additional pulses as needed for acute treatment. A significant reduction in headache frequency (33.8%), as assessed by headache diaries, was found when comparing the 28 days prior to treatment (mean of 13.3 days) to the last 28 days of treatment (mean of 8.8 days). Post-treatment data were not provided, so maintenance of effects is unknown. Improvements in headache-related disability as assessed by PedMIDAS were also found, with scores decreasing from 63 ± 46 to 27 ± 27 (57%).

### Multimodal treatment

One study assessed the effects of a multimodal treatment in adolescents with chronic tension type headache (22). The intervention was compared to a group of patients who received a preventative medication, either amitriptyline or gabapentin. The multimodal treatment group was instructed to practice complementary techniques (mindfulness and qi gong) and received osteopathic manual treatments. At 6-month follow-up, patients showed a 78% decrease in headache frequency that dropped from 22.3 to 4.9 headache days per month. Improvements were also found in secondary outcomes, such as pain intensity (67.2%), general health (67.9%), pain restriction (63%) and number of bilateral tender points (80%).

## Discussion

The results of the present literature review showed that various non-pharmacological treatments in populations of young headache patients produced sizeable effects on the primary endpoint, headache frequency, with reductions from baseline ranging between 34 and 78%. These findings are of particular interest as they are comparable to those usually found in trials on pharmacological treatments (23, 24). Moreover, many of the approaches herein reviewed produced meaningful effects on other commonly used patient-reported outcomes as well, particularly with respect to disability, quality of life, and symptoms of depression and anxiety.

The importance of considering non-pharmacological treatments in the array of possible prophylactic treatments in young headache patients lies in several factors. In particular, untoward side effects have not been reported for these procedures when applied with children and adolescents. In the rare instance when such effects have been reported for adults, they are noted to be short-lived and easily overcome (25). This stands in marked contrast to the array of side effects observed in drug prophylaxis, with the most common being sedation or somnolence, dizziness, mood/behavioral changes, constipation, increased appetite, and weight gain (6, 23). Second, in recent years these treatments—particularly behavioral ones—have gained in popularity among adult patients, while conventional pharmacological treatments are being viewed as sometimes ineffective or too expensive (26, 27). It is therefore likely that a similar trend will emerge not only among the parents of child and adolescent patients, but also among the patients themselves. Third, but no less important, non-pharmacological treatments are thought to enable young patients to enhance their abilities to handle pain and cope more effectively with pain episodes absent medications. In the long run, these learned skills may serve to reduce the risk of overusing medication as the adolescents become adults. These mentioned factors—together with the results of the present review—support the idea that non-pharmacological treatments should no longer be considered only as alternative or complementary to pharmacological treatments for headaches. Rather, they merit inclusion in the array of possible first line treatments for headache disorders, in particular among populations of children and adolescents.

Although effects are in general pronounced, mechanisms by which non-pharmacological treatments exert their effects has received only scant attention. Results from the present review suggest that, with regard to CBT and Mindfulness-based treatments in particular, headache improvement may be related in part to concurrent improvements in symptoms of anxiety and depression (14, 15, 20). In fact, available literature suggests that children and adolescents with headache disorders, and migraine in particular, may have higher symptoms of anxiety and depression when compared to healthy counterparts (28–32). We emphasize “suggestive” because of the possibility of false positive responses based on screening tools wherein certain scale items overlap some symptoms of depression, anxiety, and migraine (e.g., mood and energy level changes may incur in both premonitory and post-drome phases of migraine and are core symptoms of anxiety and depression). Taken as a whole, the conclusions of the aforementioned literature reviews indicate that the majority of young patients with headache disorders do not show diagnosable psychiatric comorbidities. However, when present, they deserve attention and appropriate treatments to improve patients' prognoses (29, 30, 33).

Headaches are regarded as bio-behavioral disorders, which means that both dysfunction in several brain areas and behavioral responses to stimuli, such as stress or pain, concur to the maintenance of the disease, which in fact may arise from the complex interaction between biological and psychosocial variables (34). The brain of patients with headache, particularly in migraineurs, is hyper-reactive to prolonged repeated stimuli, and altered inter-ictal information processing is associated with limbic system dysfunction (35). Studies specifically examining cognitive processes related to pain modulation in healthy individuals shed light on core brain regions involved in cognitive interventions, such as the prefrontal cortex, the midcingulate cortex, the thalamus, and the amygdala; i.e., the same brain areas which are involved in the cognitive and affective components of pain (36). However, these have to be taken as hypotheses, since the aforementioned studies are derived from populations of healthy adults. With regard to sTMS, it is proposed that the fluctuating magnetic field delivered by the device may induce electrical currents that disrupt cortical spreading depression (37); i.e., a wave of excitation followed by a wave of inhibition of both neurons and glia, which spreads across the cortical mantle that is purported to be a physiological substrate of migraine with aura (38). It is not associated with side effects, and it is therefore considered a safe treatment for migraine. Among adults, several studies have been carried out on both single-pulse and repetitive TMS (39–42), while the data for pediatric populations is—to the best of our knowledge—confined to the single study included in the present review. Finally, BFB is a bio-behavioral approach through which patients learn to voluntarily modify their bodily reactions via feedback-mediated awareness of physiologic parameters, such as peripheral skin temperature or electromyography (43). It is deemed to act, in part, by reducing cortical excitability and affecting resonance and oscillations of essential feedback loops in the brain (44) induced by modifications of bodily reactions through feedback-mediated awareness of physiologic parameters.

The evidence generated by the present review needs to be tempered somewhat due to certain shortcomings in the available studies, all of which need to be addressed in future research in the field of non-pharmacological treatments for pediatric headache in young patients. Many of the results herein reported have in fact been derived from single group open-label outcome studies, which preclude us from addressing comparative efficacy of these treatments. The inability to implement double-blinding for behavioral treatments remains a contentious issue for some. Although this concern cannot be addressed fully, rigor can be enhanced by randomizing participants to study and control or comparison groups and blinding those in charge of selecting, assigning, and evaluating treatment outcomes. With studies on non-invasive neurostimulation, sham procedures can be employed to enable double-blinding. Also, headache frequency was not always employed as the primary endpoint, which is specified as critical in all existing trial guidelines. Two studies reported only descriptive baseline information on headache frequency, while two studies did not report frequency at all, thus relying on measures that are traditionally employed as secondary endpoints, such as disability or quality of life. Duration of follow-up is another critical element, as most of the studies reviewed herein that reported data collection beyond the end of treatment did so only for a few months (e.g., around 3–4). This leaves us unable to draw any meaningful conclusions about stability of effects over the long term. Finally, future studies need to examine factors that mediate and/or are associated with positive outcomes. This can be accomplished in a number of ways, such as addressing changes in biomarkers associated with non-pharmacological approaches, based on neuroimaging and biological assays, and developing predictive models. In fact patient selection is of paramount importance in pediatric populations, and thus future studies should encompass a wide spectrum of clinical, psychosocial and biological indicators, in order to identify which are the most relevant patient features that are associated with positive clinical changes.

Our search was confined to SCOPUS because its search engine is noted to be wide ranging and journals within it are indexed from both medical and social science fields. Further, great care was taken to employ quality control measures aimed to reduce the possibility that relevant papers were excluded. Nonetheless, we cannot be certain that all relevant articles were included in our review process. Given our resultant small sample size, overlooking just a few salient articles may have altered our conclusions. Nutraceuticals, another prominent area of non-pharmacological treatments, were not included in the present review because an extensive literature review of this domain was published at the time we launched our search (45). This more recent review confirmed the results of previous current reviews (46, 47); i.e., that few studies exist, most are of low quality, and, consequently, the evidence generated thus far remains sparse.

## Conclusions

Our review on the use of non-pharmacological approaches in young patients with primary headaches showed that these treatments produced sizeable effects on headache frequency, with reductions from baseline ranging between 34 and 78%, which in fact is comparable to that obtained when treating patients with pharmacological compounds. When reported, these treatments led to positive outcomes in various secondary endpoints as well.

Our findings reinforce the conclusions expressed by authors of other recent literature reviews (6, 7). We share the opinion that preventive drug treatment for headache is not always needed in young headache patients, and that the risk of side effects must always be taken into account. Conversely, clinicians should consider non-pharmacological treatments of headache disorders as a first line strategy in children and adolescents with primary headaches.

Future studies, incorporating random assignment, relying on headache frequency as the primary endpoint, employing more extended follow-up periods, and assessing possible mechanisms of treatment, such as changes in relevant biomarkers, would help to shore up the existing data base for the overall value of non-pharmacological treatments for children and adolescents experiencing recurrent headache. Determining factors predictive of outcome merits intensive study as well.

## Author contributions

FA, LG, and DD led the initiative and revised the drafted document. ES selected abstracts and revised the drafted document. AR and EG selected abstract, extracted data and drafted the manuscript. All authors approved the final version.

### Conflict of interest statement

The authors declare that the research was conducted in the absence of any commercial or financial relationships that could be construed as a potential conflict of interest.
