# Supplementary material for: Non-pharmacological Approaches for Headaches in Young Age: An Updated Review
Source: Front Neurol. 2018 Nov 27;9:1009. doi: 10.3389/fneur.2018.01009 (PMC6277635; doi:10.3389/fneur.2018.01009)
Supplement: Supplementary file 1 [file Table_1.DOCX]

**Search Strategy (performed on July 23, 2018)**

( ( TITLE-ABS-KEY ( young OR child* OR adolesc* OR juvenile ) ) AND ( TITLE-ABS-KEY ( headache* OR "tension type headache" OR migraine OR "chronic tension type headache" OR "chronic migraine" OR "medication overuse headache" ) ) AND ( ( TITLE-ABS-KEY ( "cognitive behavio*" OR "Acceptance and commitment therapy" OR act OR mindfulness OR biofeedback OR "relaxation training" OR "lifestyle modification*" OR "complementary alternative medicine" ) OR TITLE-ABS-KEY ( neuromodulation OR neurostimulation OR "single pulse transcranial magnetic stimulation" OR "repetitive transcranial magnetic stimulation" OR "transcutaneous supraorbital nerve stimulation" OR "non invasive vagal nerve stimulation" ) OR TITLE-ABS-KEY ( "caloric vestibular stimulation" OR "sphenopalatine ganglion stimulation" OR "occipital nerve stimulation" ) ) ) ) AND ( LIMIT-TO ( DOCTYPE , "ar " ) OR LIMIT-TO ( DOCTYPE , " ar " ) ) AND ( LIMIT-TO ( SUBJAREA , "MEDI " ) OR LIMIT-TO ( SUBJAREA , " PHAR " ) OR LIMIT-TO ( SUBJAREA , " BIOC " ) OR LIMIT-TO ( SUBJAREA , " NEUR " ) OR LIMIT-TO ( SUBJAREA , " PSYC " ) OR LIMIT-TO ( SUBJAREA , " HEAL " ) OR LIMIT-TO ( SUBJAREA , " MEDI " ) OR LIMIT-TO ( SUBJAREA , " NEUR " ) OR LIMIT-TO ( SUBJAREA , " PSYC " ) OR LIMIT-TO ( SUBJAREA , " BIOC " ) OR LIMIT-TO ( SUBJAREA , " PHAR " ) OR LIMIT-TO ( SUBJAREA , " HEAL " ) OR EXCLUDE ( SUBJAREA , " IMMU " ) OR EXCLUDE ( SUBJAREA , " AGRI " ) OR EXCLUDE ( SUBJAREA , " ENVI " ) OR EXCLUDE ( SUBJAREA , " SOCI " ) OR EXCLUDE ( SUBJAREA , " ARTS " ) OR EXCLUDE ( SUBJAREA , " VETE " ) OR EXCLUDE ( SUBJAREA , " CENG " ) OR EXCLUDE ( SUBJAREA , " CHEM " ) OR EXCLUDE ( SUBJAREA , " AGRI " ) OR EXCLUDE ( SUBJAREA , " ARTS " ) OR EXCLUDE ( SUBJAREA , " ENVI " ) OR EXCLUDE ( SUBJAREA , " MATH " ) OR EXCLUDE ( SUBJAREA , " NURS " ) OR EXCLUDE ( SUBJAREA , " SOCI " ) ) AND ( LIMIT-TO ( PUBYEAR , 2018 ) OR LIMIT-TO ( PUBYEAR , 2017 ) OR LIMIT-TO ( PUBYEAR , 2016 ) OR LIMIT-TO ( PUBYEAR , 2015 ) OR LIMIT-TO ( PUBYEAR , 2014 ) OR LIMIT-TO ( PUBYEAR , 2013 ) OR LIMIT-TO ( PUBYEAR , 2012 ) OR LIMIT-TO ( PUBYEAR , 2011 ) OR LIMIT-TO ( PUBYEAR , 2010 ) OR LIMIT-TO ( PUBYEAR , 2018 ) ) AND ( EXCLUDE ( EXACTSRCTITLE , "Antimicrobial Agents And Chemotherapy " ) OR EXCLUDE ( EXACTSRCTITLE , " American Journal Of Cardiovascular Drugs " ) OR EXCLUDE ( EXACTSRCTITLE , " Antiviral Therapy " ) OR EXCLUDE ( EXACTSRCTITLE , " Clinical Infectious Diseases " ) OR EXCLUDE ( EXACTSRCTITLE , " Contraception " ) OR EXCLUDE ( EXACTSRCTITLE , " European Journal Of Drug Metabolism And Pharmacokinetics " ) OR EXCLUDE ( EXACTSRCTITLE , " Human Reproduction " ) OR EXCLUDE ( EXACTSRCTITLE , " Journal Of Cardiovascular Pharmacology " ) OR EXCLUDE ( EXACTSRCTITLE , " Journal Of Korean Neurosurgical Society " ) OR EXCLUDE ( EXACTSRCTITLE , " Lancet Infectious Diseases " ) OR EXCLUDE ( EXACTSRCTITLE , " Medico Legal Update " ) OR EXCLUDE ( EXACTSRCTITLE , " Pediatric Blood And Cancer " ) OR EXCLUDE ( EXACTSRCTITLE , " Respiratory Medicine " ) OR EXCLUDE ( EXACTSRCTITLE , " Acta Ophthalmologica " ) OR EXCLUDE ( EXACTSRCTITLE , " Aerospace Medicine And Human Performance " ) OR EXCLUDE ( EXACTSRCTITLE , " Allergologia Et Immunopathologia " ) OR EXCLUDE ( EXACTSRCTITLE , " Annals Of Emergency Medicine " ) OR EXCLUDE ( EXACTSRCTITLE , " Annals Of General Psychiatry " ) OR EXCLUDE ( EXACTSRCTITLE , " Asian Pacific Journal Of Tropical Disease " ) OR EXCLUDE ( EXACTSRCTITLE , " BMC Anesthesiology " ) OR EXCLUDE ( EXACTSRCTITLE , " BMC Infectious Diseases " ) OR EXCLUDE ( EXACTSRCTITLE , " BMC Medical Genetics " ) OR EXCLUDE ( EXACTSRCTITLE , " BMC Public Health " ) OR EXCLUDE ( EXACTSRCTITLE , " BMC Pulmonary Medicine " ) OR EXCLUDE ( EXACTSRCTITLE , " Basic And Clinical Pharmacology And Toxicology " ) OR EXCLUDE ( EXACTSRCTITLE , " Brain Injury " ) OR EXCLUDE ( EXACTSRCTITLE , " Cardiovascular Therapeutics " ) OR EXCLUDE ( EXACTSRCTITLE , " Central Nervous System Agents In Medicinal Chemistry " ) OR EXCLUDE ( EXACTSRCTITLE , " Clinical And Experimental Dermatology " ) OR EXCLUDE ( EXACTSRCTITLE , " Clinical And Experimental Nephrology " ) OR EXCLUDE ( EXACTSRCTITLE , " Clinical Cancer Research " ) OR EXCLUDE ( EXACTSRCTITLE , " Clinical Pharmacokinetics " ) OR EXCLUDE ( EXACTSRCTITLE , " Clinical Rhinology " ) OR EXCLUDE ( EXACTSRCTITLE , " Clinical Therapeutics " ) OR EXCLUDE ( EXACTSRCTITLE , " Diabetes Obesity And Metabolism " ) OR EXCLUDE ( EXACTSRCTITLE , " Drug Design Development And Therapy " ) OR EXCLUDE ( EXACTSRCTITLE , " Drug Research " ) OR EXCLUDE ( EXACTSRCTITLE , " Drug Safety " ) OR EXCLUDE ( EXACTSRCTITLE , " Drugs And Therapy Perspectives " ) OR EXCLUDE ( EXACTSRCTITLE , " Epilepsy Research " ) OR EXCLUDE ( EXACTSRCTITLE , " Epileptic Disorders " ) OR EXCLUDE ( EXACTSRCTITLE , " European Archives Of Oto Rhino Laryngology " ) OR EXCLUDE ( EXACTSRCTITLE , " European Psychiatry " ) OR EXCLUDE ( EXACTSRCTITLE , " Indian Journal Of Hematology And Blood Transfusion " ) OR EXCLUDE ( EXACTSRCTITLE , " Infectious Diseases Of Poverty " ) OR EXCLUDE ( EXACTSRCTITLE , " International Journal For Equity In Health " ) OR EXCLUDE ( EXACTSRCTITLE , " International Urology And Nephrology " ) OR EXCLUDE ( EXACTSRCTITLE , " Iranian Journal Of Psychiatry And Behavioral Sciences " ) OR EXCLUDE ( EXACTSRCTITLE , " Journal Of Affective Disorders " ) OR EXCLUDE ( EXACTSRCTITLE , " Journal Of Applied Research " ) OR EXCLUDE ( EXACTSRCTITLE , " Journal Of Clinical Endocrinology And Metabolism " ) OR EXCLUDE ( EXACTSRCTITLE , " Journal Of Clinical Psychopharmacology " ) OR EXCLUDE ( EXACTSRCTITLE , " Journal Of Clinical Rheumatology " ) OR EXCLUDE ( EXACTSRCTITLE , " Journal Of Cutaneous Medicine And Surgery " ) OR EXCLUDE ( EXACTSRCTITLE , " Journal Of Emergency Medicine " ) OR EXCLUDE ( EXACTSRCTITLE , " Journal Of Infection " ) OR EXCLUDE ( EXACTSRCTITLE , " Journal Of Neurosurgery Spine " ) OR EXCLUDE ( EXACTSRCTITLE , " Journal Of Pediatric Gastroenterology And Nutrition " ) OR EXCLUDE ( EXACTSRCTITLE , " Journal Of The European Academy Of Dermatology And Venereology " ) OR EXCLUDE ( EXACTSRCTITLE , " Journal Of Traditional Chinese Medicine " ) OR EXCLUDE ( EXACTSRCTITLE , " Lancet Oncology " ) OR EXCLUDE ( EXACTSRCTITLE , " Lancet Respiratory Medicine " ) OR EXCLUDE ( EXACTSRCTITLE , " Malaysian Journal Of Pathology " ) OR EXCLUDE ( EXACTSRCTITLE , " Medical Hypotheses " ) OR EXCLUDE ( EXACTSRCTITLE , " Medical Journal Armed Forces India " ) OR EXCLUDE ( EXACTSRCTITLE , " Medical Principles And Practice " ) OR EXCLUDE ( EXACTSRCTITLE , " Medicinal Chemistry " ) OR EXCLUDE ( EXACTSRCTITLE , " Military Medicine " ) OR EXCLUDE ( EXACTSRCTITLE , " Neuro Oncology " ) OR EXCLUDE ( EXACTSRCTITLE , " Obstetrics And Gynecology " ) OR EXCLUDE ( EXACTSRCTITLE , " Pharmacogenetics And Genomics " ) OR EXCLUDE ( EXACTSRCTITLE , " Pharmacological Research " ) OR EXCLUDE ( EXACTSRCTITLE , " Pharmacology " ) OR EXCLUDE ( EXACTSRCTITLE , " Pharmacology Research And Perspectives " ) OR EXCLUDE ( EXACTSRCTITLE , " Pulmonary Pharmacology And Therapeutics " ) OR EXCLUDE ( EXACTSRCTITLE , " Research In Social And Administrative Pharmacy " ) OR EXCLUDE ( EXACTSRCTITLE , " Reviews On recent Clinical Trials " ) OR EXCLUDE ( EXACTSRCTITLE , " Seizure " ) OR EXCLUDE ( EXACTSRCTITLE , " Therapeutic Innovation And Regulatory Science " ) OR EXCLUDE ( EXACTSRCTITLE , " World Neurosurgery " ) ) AND ( LIMIT-TO ( LANGUAGE , "English " ) OR LIMIT-TO ( LANGUAGE , " English " ) OR EXCLUDE ( LANGUAGE , " German " ) OR EXCLUDE ( LANGUAGE , " Turkish " ) OR EXCLUDE ( LANGUAGE , " French " ) OR EXCLUDE ( LANGUAGE , " Spanish " ) )
